# Enhanced character-level deep convolutional neural networks for cardiovascular disease prediction

**DOI:** 10.1186/s12911-020-1118-z

**Published:** 2020-07-09

**Authors:** Zhichang Zhang, Yanlong Qiu, Xiaoli Yang, Minyu Zhang

**Affiliations:** 1 College of Computer Science and Engineering, Northwest Normal University, 967 Anning East Road, Lanzhou, 730070 China; 2 College of Computer Science and Engineering, Northwest Normal University, 967 Anning East Road, Lanzhou, 730070 China; 3 College of Computer Science and Engineering, Northwest Normal University, 967 Anning East Road, Lanzhou, 730070 China; 4 College of Computer Science and Engineering, Northwest Normal University, 967 Anning East Road, Lanzhou, 730070 China

**Keywords:** Chinese electronic medical record, CVD risk factors extraction, CVD prediction, Downsampling, Pre-activation, Dimension-matching free, Text region embedding

## Abstract

**Background:**

Electronic medical records contain a variety of valuable medical information for patients. So, when we are able to recognize and extract risk factors for disease from EMRs of patients with cardiovascular disease (CVD), and are able to use them to predict CVD, we have the ability to automatically process clinical texts, resulting in an improved accuracy of supporting doctors for the clinical diagnosis of CVD. In the case where CVD is becoming more worldwide, predictive CVD based on EMRs has been studied by many researchers to address this important aspect of improving diagnostic efficiency.

**Methods:**

This paper proposes an Enhanced Character-level Deep Convolutional Neural Networks (EnDCNN) model for cardiovascular disease prediction.

**Results:**

On the manually annotated Chinese EMRs corpus, our risk factor identification extraction model achieved 0.9073 of F-score, our prediction model achieved 0.9516 of F-score, and the prediction result is better than the most previous methods.

**Conclusions:**

The character-level model based on text region embedding can well map risk factors and their labels as a unit into a vector, and downsampling plays a crucial role in improving the training efficiency of deep CNN. What’s more, the shortcut connections with pre-activation used in our model architecture implements dimension-matching free in training.

## Background

CVD is becoming more common worldwide and is becoming younger. According to data released by the World Health Organization, CVD is the number one cause of death worldwide, with more deaths from cardiovascular disease each year than any other cause of death. In 2016, an estimated 17.9 million people died of cardiovascular disease, accounting for 31% of all deaths worldwide. In its 2018 report, China’s National Center for Cardiovascular Disease noted that CVD mortality remained at the top of 2016, higher than cancer and other diseases, and the number of patients was as high as 290 million.

As CVD risk increases in China, interest in strategies to mitigate it is growing. However, information on the prevalence and treatment of CVD in daily life is limited. But in the medical field, many hospitals have been able to systematically accumulate medical records for a large number of patients by introducing an EMR system. Deep learning has been successfully applied to medical field based on accumulated EMR data [1, 2]. In particular, many studies have been conducted to predict the risk of cardiovascular disease in order to prevent cardiovascular diseases with a high mortality rate globally [3]. Because EMR data is recorded based on patients visiting the hospital, and it contains information on the pathogenesis of cardiovascular disease. Therefore, we intend to extract key information using Convolutional Neural Networks (CNN). Table 1 is the key information we consider, including twelve risk factors. However, we found that there was a large amount of irrelevant information in most EMRs. For example, a record of a complete medical record contains only 10 records of information that is effective in causing disease. These excessively irrelevant information not only reduces CNN’s emphasis on effective disease information, but also greatly delays the training time of neural networks. In this regard, we propose to extract the risk factors that cause disease in EMRs and bring along the Time Attribute of these risk factors. In fact, although the training time has decreased, the experimental results are not optimistic. After experimental analysis, we believe that the main reason is the lack of certain context information. In response to this situation, we proposed the EnDCNN. The region embedding method used by EnDCNN can enhance the correlation between risk factors. For example, an increased correlation between the risk of hypertension and the risk of controlling blood pressure can better predict whether the patient has heart disease. At the same time, our inspiration from the ResNet network proposed by He et al. [4] has deepened our own neural network to better extract key information. And for our deep CNN model features, we also have the downsampling method to further reduce training time. This makes it not only speed up the training time through our method, but also the experimental result F-score reaches 0.9516, which fully demonstrates the method we proposed and the efficiency of the network architecture we took out. In summary, our contribution is two-fold, which can be concluded as follows:

**Table 1:** Attributes of CVD

**No.**	**Attributes**	**Description**
1.	Overweight/Obesity (O2)	A diagnosis of patient overweight or obesity
2.	Hypertension	A diagnosis or history of hypertension
3.	Diabetes	A diagnosis or a history of diabetes
4.	Dyslipidemia	A diagnosis of dyslipidemia, hyperlipidemia or
		a history of hyperlipidemia
5.	Chronic Kidney Disease (CKD)	A diagnosis of CKD
6.	Atherosis	A diagnosis of atherosclerosis or atherosclerotic plaque
7.	Obstructive Sleep Apnea Syndrome (OSAS)	A diagnosis of OSAS
8.	Smoking	Smoking or a patient history of smoking
9.	Alcohol Abuse (A2)	Alcohol abuse
10.	Family History of CVD (FHCVD)	Patient has a family history of CVD or has a first-degree relative
		(parents,siblings, or children) who has a history of CVD
11.	Age	The age of the patient
12.	Gender	The gender of patient

Our innovation proposes to extract the risk factor identification and bring along its corresponding label as the basis for CVD prediction. Recurrent Neural Networks (RNNs) generally read the whole text from beginning to end or vice versa sometimes, which makes it inefficient to process long texts [5]. In this regard, Huang et al. deal with Long Short-Term Memory (LSTM) in English text. In view of this, we propose to extract the risk factors and their corresponding labels recognition for the characteristics of the CNN network we use. This method not only avoids a large amount of non-critical information, but also reduces the time spent on model architecture training to some extent.

We propose the EnDCNN model architecture. Inspired by the application of region embedding by Johnson et al. [6] and the ResNet for image model architecture [4] proposed by He et al. We first convert the risk factors and their corresponding tags into corresponding vectors in characters by our character embedding trained in a specific field. Then, we built deep CNN to better extract key information. Finally, for our deep model architecture, we used the downsampling method to further speed up the training, and the final effect of the model is optimistic.

## Methods

**Fig. 1:**
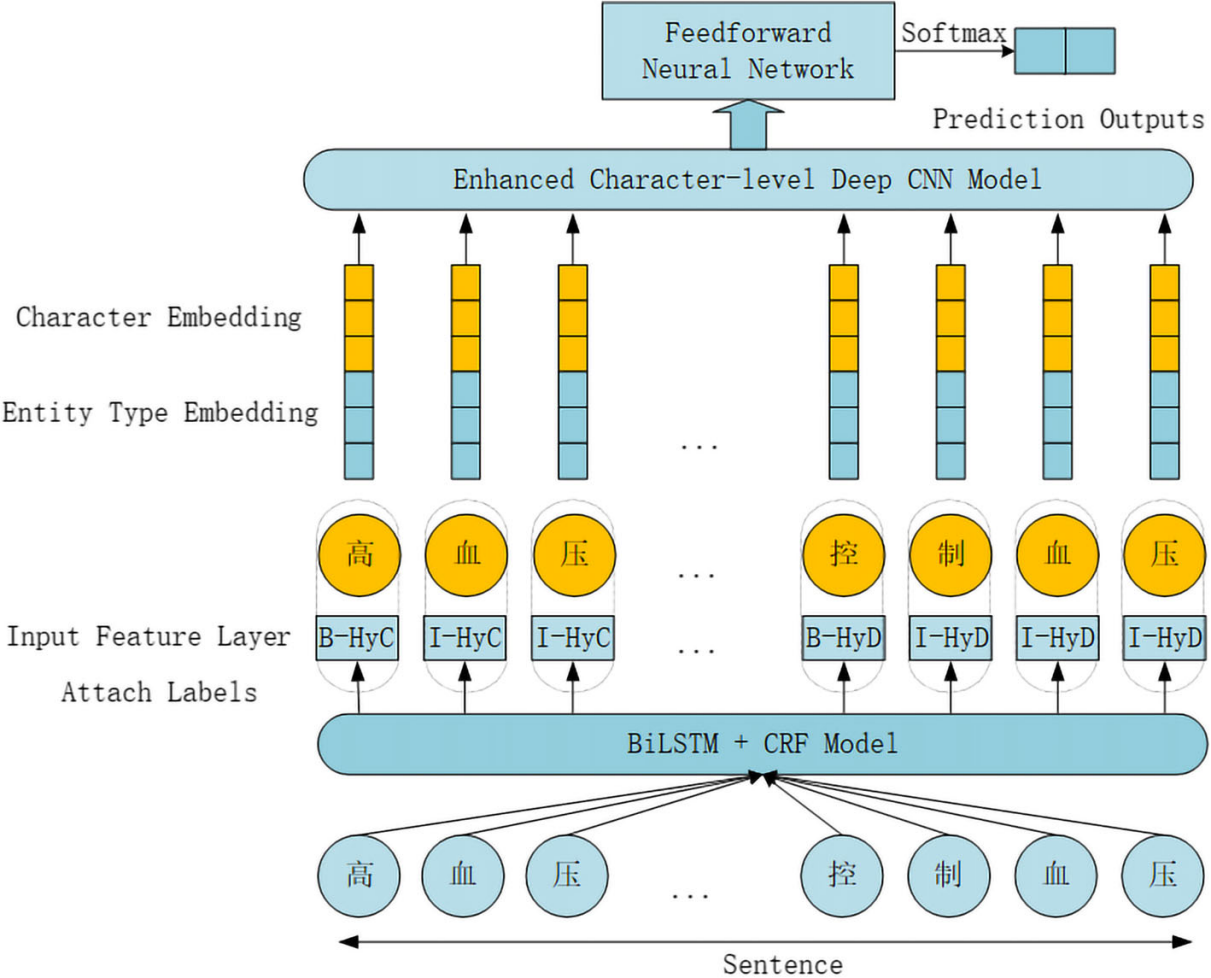
The entire model architecture of our proposed

The main idea of this paper is to predict whether a patient has CVD by focusing on the risk factors in EMRs. First of all, we need to prepare the data we need. The user enters the appropriate input values from his/her EMR report. After this, the historical dataset is uploaded. The fact that most medical dataset may contain missing values makes this accurate prediction difficult. So, for this missing data, we have to transform the missing data into structured data with the help of a data cleaning and data imputation process. After preparing the data, we mainly perform the following two steps. Firstly, the risk factors in the EMRs and their corresponding labels identification are then extracted using the relatively mature entity recognition technology that has been developed. In addition to Age and Gender, the labels for other risk factors include the type of the risk factor and its temporal attributes. We only use the Conditional Random Field (CRF) layer to identify the F-score of the extraction result to reach 0.8994. When we use bidirectional LSTM with a CRF layer (BiLSTM-CRF), the F-score identifying the extraction results reached 0.9073. We did a lot of experiments and summarized why there is such a high F-score based on experimental and EMRs data analysis. Because there are 12 risk factors in the entire data, these risk factors are largely repeated in EMRs. This is great for the system we have proposed. In contrast, the BiLSTM-CRF model has better recognition performance, so we consider using it to extract the risk factors and corresponding labels in EMRs. These extracted risk factors that carry the corresponding labels serve as the basis for input and predict CVD. In the end, by using the EnDCNN model architecture, we can predict whether a patient has CVD. Figure 1 shows our entire model architecture.

### BiLSTM-CRF

**Fig. 2:**
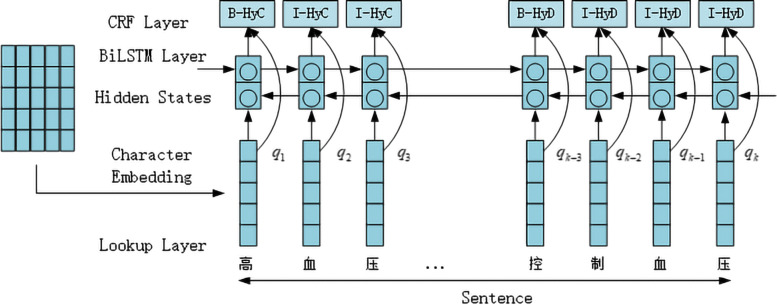
The architecture of BiLSTM-CRF model

The architectures of BiLSTM-CRF model illustrated in Fig. 2. In the model, the BIO (Begin, Inside, Outside) tagging scheme is used.The **Q**=(**q**_1_,...,**q**_*k*−3_,...,**q**_*k*_) represents the context information carried by the character embedding trained by Skip-Gram.The HyC is represented as Hypertension and its temporal attribute is *Continue*. The HyD is represented as Hypertension and its temporal attribute is *During*. It is similar to the ones presented by Huang et al. [7], Lample et al. [8] and Ma and Hovy [9].

Given a sentence, the model predicts a label corresponding to each of the input tokens in the sentence. Firstly, through the embedding layer, the sentence is represented as a sequence of vectors **X**=(**x**_1_,...,**x**_*t*_,...,**x**_*n*_) where n is the length of the sentence. Next, the embeddings are given as input to a BiLSTM layer. In the BiLSTM layer, a forward LSTM computes a representation $\stackrel {\rightarrow }{\textbf {h}_{t}}$ of the sequence from left to right at every character t, and another backward LSTM computes a representation $\stackrel {\leftarrow }{\textbf {h}_{t}}$ of the same sequence in reverse. These two distinct networks use different parameters, and then the representation of a character $\textbf {h}_{t}=\left [\stackrel {\rightarrow }{\textbf {h}_{t}};\stackrel {\leftarrow }{\textbf {h}_{t}}\right ]$ is obtained by concatenating its left and right context representations. LSTM memory cell is implemented as Lample et al. [8] did.

Then a tanh layer on top of the BiLSTM is used to predict confidence scores for the character having each of the possible labels as the output scores of the network.

1 $$\begin{array}{@{}rcl@{}} \mathbf{e}_{t}=\tanh(\mathbf{W}_{e}\mathbf{h}_{t}), \end{array} $$

where the weight matrix **W**_*e*_ is the parameter of the model to be learned in training.

Finally, instead of modeling tagging decisions independently, the CRF layer is added to decode the best tag path in all possible tag paths. We consider **P** to be the matrix of scores output by the network. The *t*^*th*^ column is the vector *e*_*t*_ obtained by the Equation (1). The element *P*_*i*,*j*_ of the matrix is the score of the *j*^*th*^ tag of the *i*^*th*^ character in the sentence. We introduce a tagging transition matrix **T**, where *T*_*i*,*j*_ represents the score of transition from tag *i* to tag *j* in successive characters and *T*_0,*j*_ as the initial score for starting from tag *j*. This transition matrix will be trained as the parameter of model. The score of the sentence **X** along with a sequence of predictions **y**=(*y*_1_,...,*y*_*t*_,...,*y*_*n*_) is then given by the sum of transition scores and network scores:

2 $$\begin{array}{@{}rcl@{}} s(\mathbf{X},\mathbf{y}) = \sum\limits_{i=1}^{N}\left(T_{y_{i-1},y_{i}}+P_{i,y_{i}}\right), \end{array} $$

Then a softmax function is used to yield the conditional probability of the path **y** by normalizing the above score over all possible tag paths $\tilde {\mathbf {y}}$:

3 $$\begin{array}{@{}rcl@{}} p\left(\mathbf{y}|\mathbf{X}\right) = \frac{e^{s\left(\mathbf{X},\mathbf{y}\right)}}{\sum_{\tilde{\mathbf{y}}}e^{s}\left(\mathbf{X},\tilde{\mathbf{y}}\right)}, \end{array} $$

During the training phase, the objective of the model is to maximize the log-probability of the correct tag sequence. At inference time, we predict the best tag path that obtains the maximum score given by:

4 $$\begin{array}{@{}rcl@{}} \arg_{\tilde{\mathbf{y}}} \max s\left(\mathbf{X},\tilde{\mathbf{y}}\right), \end{array} $$

This can be computed using dynamic programming, and the Viterbi algorithm [10] is chosen for this inference.

### Overview of EnDCNN

**Fig. 3:**
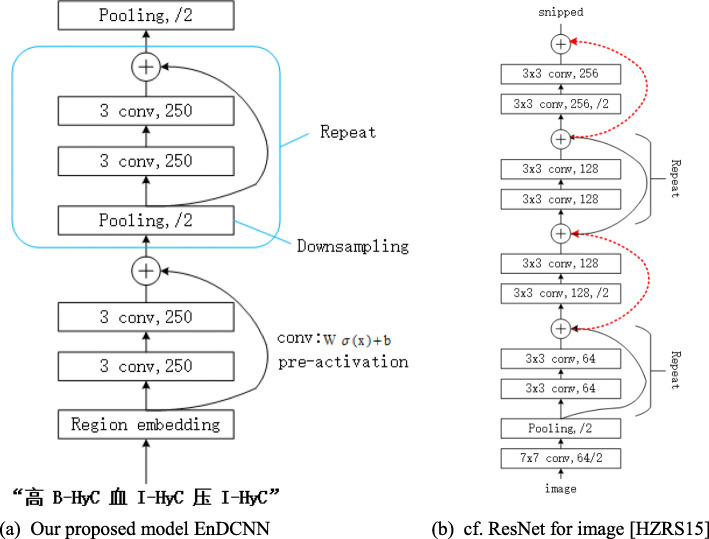
The architectures of our model and ResNet

Figure 3a is our proposed model EnDCNN. Figure 3b is He et al. proposed the ResNet network architecture for image. ⊕ indicates addition. The dotted red shortcuts in Fig. 3b perform dimension matching. EnDCNN is dimension-matching free. The first layer of our model performs text region embedding, which generalizes commonly used character embedding to the embedding of text regions covering one or more characters. It is followed by stacking of convolution blocks (two convolution layers and a shortcut) interleaved with pooling layers with stride 2 for downsampling. The final pooling layer aggregates internal data for each document into one vector. We use max pooling for all pooling layers. The key features of EnDCNN are as follows.

Downsampling without increasing the number of feature maps (dimensionality of layer output, 250 in Fig. 3a). Downsampling enables efficient represent-ation of long-range associations (and so more global information) in the text. By keeping the same number of feature maps, every 2-stride downsampling reduces the per-block computation by half and thus the total computation time is bounded by a constant.

Shortcut connections with pre-activation and identity mapping [11] for enabling training of deep networks.

Text region embedding enhances the relevance of individual character to character, the relevance of risk factors to their corresponding tags. When we use risk factors and their corresponding labels as a unit, text region embedding can also enhance the correlation between each unit. Therefore, accuracy can be improved by text region embedding.

### Network architecture of EnDCNN

**Downsampling with the number of feature maps fixed** After each convolution block, we perform max-pooling with size 3 and stride 2. That is, the pooling layer produces a new internal representation of a document by taking the component-wise maximum over 3 contiguous internal vectors, representing 3 overlapping text regions, but it does this only for every other possible triplet (stride 2) instead of all the possible triplets (stride 1). This 2-stride downsampling reduces the size of the internal representation of each document by half.

A number of models (Simonyan and Zisserman, 2015 [12]; He et al., 2015 [13], 2016 [11]; Conneau et al., 2016 [14]) increase the number of feature maps whenever downsampling is performed, causing the total computational complexity to be a function of the depth. In contrast, we fix the number of feature maps, as we found that increasing the number of feature maps only does harm - increasing computation time substantially without accuracy improvement, as shown later in the experiments. With the number of feature maps fixed, the computation time for each convolution layer is halved (as the data size is halved) whenever 2-stride downsampling is performed. Therefore, with EnDCNN, the total computation time is bounded by a constant - twice the computation time of a single block, which makes our deep networks computationally attractive.

In addition, downsampling with stride 2 essentially doubles the effective coverage (i.e., coverage in the original document) of the convolution kernel; therefore, after going through downsampling *L* times, associations among characters within a distance in the order of 2^*L*^ can be represented. Thus, deep CNN is computationally efficient for representing long-range associations and so more global information.

**Shortcut connections with pre-activation** To enable training of deep networks, we use additive shortcut connections with identity mapping, which can be written as **z**+*f*(**z**) where *f* represents the skipped layers [11]. In EnDCNN, the skipped layers *f*(**z**) are two convolution layers with *pre-activation*. Here, pre-activation refers to activation being done *before* weighting instead of *after* as is typically done. That is, in the convolution layer of EnDCNN, **W***σ*(**x**)+**b** is computed at every location of each document where a column vector **x** represents a small region (overlapping with each other) of input at each location, *σ*(·) is a component-wise nonlinear activation, and weights **W** and biases **b** (unique to each layer) are the parameters to be trained. The number of **W**’s rows is the *number of feature maps* (also called the *number of filters* [13]) of this layer.We set activation *σ*(·) to the rectifier *σ*(*x*)=*max*(*x*,0). In our implementation, we fixed the number of feature maps to 250 and the kernel size (the size of the small region covered by x) to 3, as shown in Fig. 3a.

With pre-activation, it is the results of linear weighting **W***σ*(**x**)+**b** that travel through the shortcut, and what is added to them at a ⊕ (Fig. 3a) is also the results of linear weighting, instead of the results of nonlinear activation (*σ*(**Wx**+**b**)). Intuitively, such ’linearity’ eases training of deep networks, similar to the role of constant error carousels in LSTM [15]. We empirically observed that pre-activation indeed outperformed ’post-activation’, which is in line with the image results [11].

**No need for dimension matching** Although the shortcut with pre-activation was adopted from the *improved ResNet* of [11], our model is simpler than ResNet (Fig. 3b), as all the shortcuts are exactly simple *identity mapping* (i.e., passing data exactly as it is) without any complication for dimension matching. When a shortcut meets the ’main street’, the data from two paths need to have the same dimensionality so that they can be added; therefore, if a shortcut skips a layer that changes the dimensionality, e.g., by downsampling or by use of a different number of feature maps, then a shortcut must perform dimension matching. Dimension matching for increased number of feature maps, in particular, is typically done by projection, introducing more weight parameters to be trained. We eliminate the complication of dimension matching by not letting any shortcut skip a downsampling layer, and by fixing the number of feature maps throughout the network. The latter also substantially saves computation time as mentioned above, and we will show later in our experiments that on our tasks, we do not sacrifice anything for such a substantial efficiency gain.

### Text region embedding for EnDCNN

A CNN for disease prediction typically starts with converting each character in the text to a character vector (character embedding). There is no exception in our mission, we need to put each Chinese character and its corresponding label. This is the entity type embedding layer and character embedding layer in Fig. 1. Then, we take a more general viewpoint as in [16] and consider text region embedding - embedding of a region of text covering one or more characters.

In the region embedding layer we compute **Wx**+**b** for each character of a document where input **x** represents a *k*-character region (i.e., window) around the character in some straightforward manner, and weights **W** and bias **b** are trained with the parameters of other layers. Activation is delayed to the pre-activation of the next layer. Now let *v* be the size of vocabulary, and let us consider the types of straightforward representation of a *k*-character region for **x**. We chose sequential input: the *kv*-dimensional concatenation of *k* one-hot vectors.

A region embedding layer with region size *k*>1 seeks to capture more complex concepts than single characters in one weight layer, whereas a network with character embedding uses *multiple* weight layers to do this, e.g., character embedding followed by a convolution layer. Because we want to enter the risk factor and bring along its corresponding label, such as “ B-HyC”, we adjust the parameter *k* in units of 7 according to the actual situation of the experimental data.

## Results

### Dataset and evaluation metrics

**Fig. 4:**
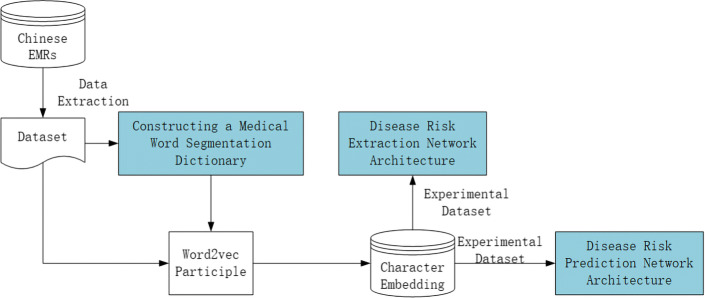
Generate the character embedding for experiments

Our dataset contains two corpora. The first corpus came from a hospital in Gansu Province with 800,000 unlabeled EMRs of internal medicine. The dataset was mainly used to train and generate our character embedding. In Fig. 4, we also added a dictionary of risk factors during the training. In this way, the Skip-Gram model in word2vec we use can better make each character in the risk factor more compact. The other one is from the Network Intelligence Research Laboratory of the Language Technology Research Center of the School of Computer Science, Harbin Institute of Technology, which contains 1186 EMRs. This corpus intends to be used to develop a risk factor information extraction system that, in turn, can be applied as a foundation for the further study of the progress of risk factors and CVD [17]. For the corpus, we divided it into CVD and no CVD according to the clinically diagnosed disease in the electronic medical record. In the corpus we used, there were 527 EMRs and 132 EMRs. The basis comes from the following two parts: On the one hand, according to the definition of CVD by the World Health Organization[18]. On the other hand, the first (symptoms) and the third (diseases) in the book of *Clinical Practical Cardiology* [19].

Our experiments involve 12 risk factors which are Overweight/Obesity (O2), Hypertension, Diabetes, Dyslipidemia, Chronic Kidney Disease (CKD), Atherosis, Obstructive Sleep Apnea Syndrome (OSAS), Smoking, Age, Gender, Abuse (A2), Family History of CVD (FHCVD), as shown in Table 1. In addition, the dataset also includes 4 temporal attributes which are *Continue*, *During*, *After*, *Before*. Since risk factors of Age and Gender have no temporal attributes, we have added a temporal attribute: None.

In dataset, it consists of unstructured data, meaning data which is not in well-formed data. Mostly medical data is not in proper format. For the missing data, imputation and data cleaning are necessary. The unwanted data and noisy data must remove from dataset so that we get structured data.

In the experiment, our training set contained 461 EMRs, the test set contained 132 EMRs, and the development set contained 66 EMRs. *Accuracy*, *Precision*, *Recall* and *F-score* are used as evaluation metrics.

### Models and parameters

We carry out the experiments to compare the performance of our model with others described in the following.

***CRF***: This model was used by Mao, et al. [20] recognized the named entity in the electronic medical records based on Conditional Random Field.

***BiLSTM-CRF***: This model was used by Li, et al. In order to realize automatic recognition and extraction of entities in unstructured medical texts, a model combining language model conditional random field algorithm (CRF) and Bi-directional Long Short-term Memory networks (BiLSTM) is proposed [21].

***SVM***: This model was used by S. Menaria, et al. As a traditional machine learning method, the support vector machine algorithm performs well in [22].

***ConvNets***: This model was used by Xiang, et al. [23] offers an empirical exploration on the use of character-level convolutional networks (ConvNets) for text classification.

***LSTM***: This model was used by Xin, et al. [24], which proposed an LSTM network with fully connected layer and activation layers.

***EnDCNN***: This is the model proposed in this paper. Table 2 gives the chosen hyper-parameters for all experiments. We tune the hyper-parameters on the development set by random search. We try to share as many hyper-parameters as possible in experiments.

**Table 2:** Hyper parameters of EnDCNN

**Parameter**	**Description**	**Value**
*d*_*w*_	Dimension of word embedding	100
*lr*	Learning rate	0.001
*B*	Batch size	64
*kp*	Each neuron’s keep rate	0.5
*dr*	Decay rate for *lr*	0.99
*ds*	Number of decay steps	500
*ks*	Window size	3
*m*	Number of filters	250
*s*	Number of strides	2
*n*	Number of epochs	30
*d*_*p*_	The depth of EnDCNN	15

### Experimental results

**Fig. 5:**
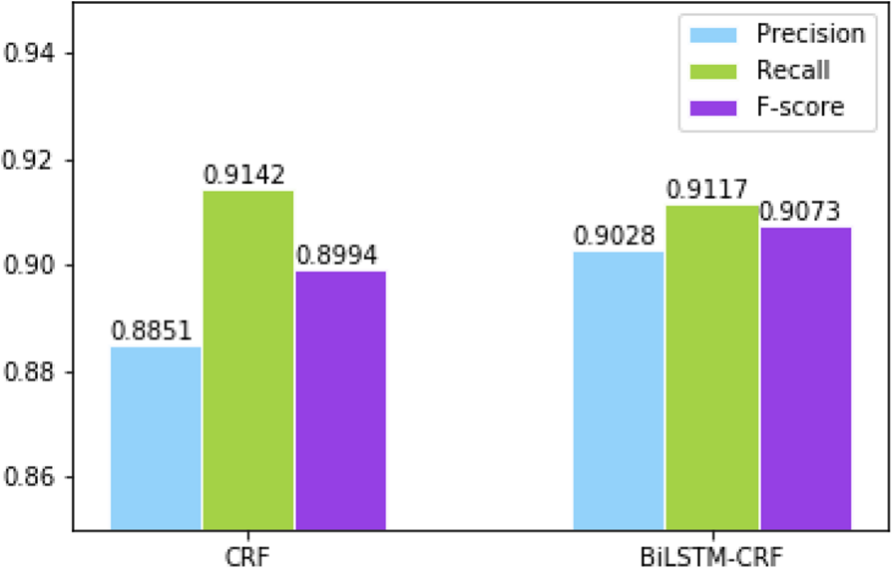
Comparison of CRF and BiLSTM-CRF models

We did a rich comparative experiment on our own model itself and other models:

In Fig. 5, we performed a comparison of the CRF and BiLSTM-CRF models for the identification of risk factors in EMRs. The precision values for the two models are: 0.8851 and 0.9028; The recall values for the two models are: 0.9142 and 0.9117; The F-score values for the two models are: 0.8994 and 0.9073, respectively. It is clear that the BiLSTM-CRF model outperforms the CRF. So, we chose the BiLSTM-CRF model as our extractor for risk factors in EMRs.

In Table 3, we show the comparison between the previous model and our proposed EnDCNN model for accuracy, precision, recall, and F-score. In addition, we also compared the performance of EnDCNN models with different ranges (*k* values) for region embedding. And the performance of each model when the dataset is the original EMRs, the risk factor with the label, or the risk factor without the label.

**Table 3:** The comparison of each model for CVD prediction results

**Model**	***k***	**Accuracy%**	**Precision%**	**Recall%**	**F-score%**
	1	93.91	93.87	93.91	93.89
*EnDCNN*	7	**95.22**	**95.14**	**95.22**	**95.16**
	14	94.35	94.31	94.35	94.32
	3	**93.91**	**93.83**	**93.91**	**93.86**
*EnDCNN*_(*nolabels*)_	4	89.57	90.19	89.57	89.87
	5	86.52	86.41	86.52	86.47
*SVM*_(*raw*)_		90.91	90.91	90.91	90.91
*SVM*_(*nolabels*)_		89.39	89.03	89.39	89.21
*ConvNets*_(*raw*)_		92.83	92.64	92.83	92.73
*LSTM*_(*raw*)_		92.24	93.46	92.73	93.09
*LSTM*_(*riskswithlabels*)_		82.58	81.35	83.01	82.17

In Table 4, we compared four cases: (1) The performance of the ConvNets model in random embedding; (2) The performance of the LSTM model in random embedding; (3) When our model is in random embedding and the region embedding size is 7; (4) The performance of our model without region embedding, that is, only use our pre-trained embedding.

**Table 4:** The performance of each model at different embedding

**Model**	**Accuracy%**	**Precision%**	**Recall%**	**F-score%**
*ConvNets*_(*nopre*−*emd*)_	91.67	90.87	91.24	91.05
*LSTM*_(*nopre*−*emd*)_	81.82	79.45	82.36	80.88
*EnDCNN*_(*nopre*−*emd*,*k*=7)_	94.13	94.06	94.13	94.09
*EnDCNN*_(*nore*−*emd*)_	88.04	87.54	88.04	87.79

**Fig. 6:**
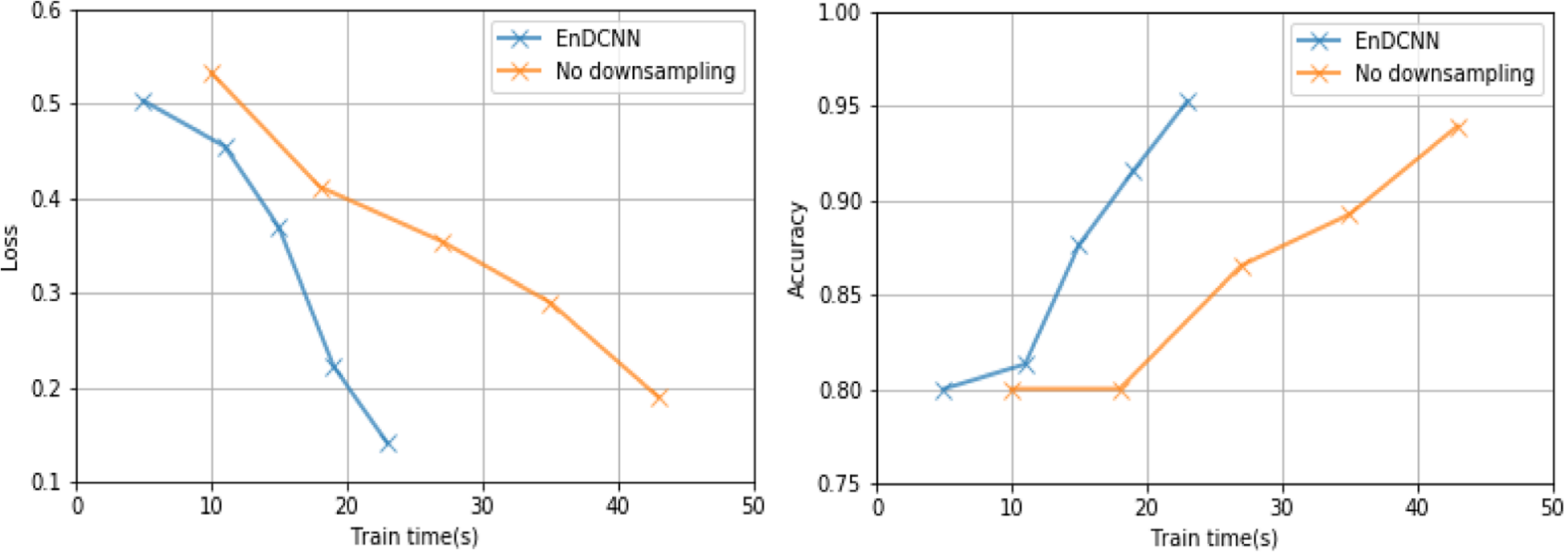
Training efficiency

In Fig. 6, we show a comparison of the training efficiencies of our model without downsampling and with downsampling. We plot the loss and accuracy in relation to the computation time - the time spent for training task using our performances on a GPU. We recorded five iterations from the start of training to the optimal training.

## Discussion

Table 3 shows the overall classification performance of different models on our evaluation corpus. It can be seen that when the region embedding range size is 7, our EnDCNN method is superior to other methods in all evaluation indicators. On the data without the risk factor labels, our model performed well when the region embedding was 3. From the performance of our model in Tables 3 and 4, we can use the character embedding pre-trained by medical EMRs to help improve the performance of the model. Not only that, but from the performance of our model in Table 4 without region embedding, the importance of region embedding to the performance of our model. For Fig. 6, we can clearly see that downsampling is critical to the training efficiency of deep convolutional neural networks like ours.

## Conclusions

In this paper, the disease prediction experiment was carried out on the EnDCNN algorithm using structured data. We used CRF and BiLSTM-CRF algorithm to identify the risk of CVD and its corresponding risk factors. We have compared the results of the CRF algorithm with the BiLSTM-CRF algorithm and the accuracy of BiLSTM-CRF 90.73% which is more than CRF algorithm. With the help of region embedding, we used the character-level embedding to achieve greater results, and the disease prediction F-score reached 95.16%. On the other hand, the downsampling technique solves the problem of slower training time in deep CNN. What’s more, the shortcut connections with pre-activation used in our model architecture implements dimension-matching free in training. In the end, we got accurate disease prediction as output, by giving the input as patients EMRs which help us to understand the level of disease prediction. This output predicted whether to have or not to have heart disease. Because of this system may leads in low time consumption and minimal cost possible for disease risk prediction. In the future, we will strengthen research on the pathogenic factors of CVD and improve the accuracy of CVD prediction as much as possible.

## Data Availability

The datasets used and analyzed during the current study are available from the corresponding author upon reasonable requests.

## Abbreviations

CVD: Cardiovascular Disease
EnDCNN: Enhanced Character-level Deep Convolutional Neural Networks
CNN: Convolutional Neural Networks
RNNs: Recurrent Neural Networks
LSTM: Long Short-Term Memory
CRF: Conditional Random Field
BiLSTM: Bi-directional Long Short-term Memory Networks
BiLSTM-CRF: Bidirectional LSTM with a CRF Layer
SVM: Support Vector Machine
ConvNets: Character-level Convolutional Networks
